# Evaluation of methods for differential expression analysis on multi-group RNA-seq count data

**DOI:** 10.1186/s12859-015-0794-7

**Published:** 2015-11-04

**Authors:** Min Tang, Jianqiang Sun, Kentaro Shimizu, Koji Kadota

**Affiliations:** Graduate School of Agricultural and Life Sciences, The University of Tokyo, 1-1-1 Yayoi, Bunkyo-ku, Tokyo, 113-8657 Japan

## Abstract

**Background:**

RNA-seq is a powerful tool for measuring transcriptomes, especially for identifying differentially expressed genes or transcripts (DEGs) between sample groups. A number of methods have been developed for this task, and several evaluation studies have also been reported. However, those evaluations so far have been restricted to two-group comparisons. Accumulations of comparative studies for multi-group data are also desired.

**Methods:**

We compare 12 pipelines available in nine R packages for detecting differential expressions (DE) from multi-group RNA-seq count data, focusing on three-group data with or without replicates. We evaluate those pipelines on the basis of both simulation data and real count data.

**Results:**

As a result, the pipelines in the TCC package performed comparably to or better than other pipelines under various simulation scenarios. TCC implements a multi-step normalization strategy (called DEGES) that internally uses functions provided by other representative packages (edgeR, DESeq2, and so on). We found considerably different numbers of identified DEGs (18.5 ~ 45.7 % of all genes) among the pipelines for the same real dataset but similar distributions of the classified expression patterns. We also found that DE results can roughly be estimated by the hierarchical dendrogram of sample clustering for the raw count data.

**Conclusion:**

We confirmed the DEGES-based pipelines implemented in TCC performed well in a three-group comparison as well as a two-group comparison. We recommend using the DEGES-based pipeline that internally uses edgeR (here called the *EEE-E* pipeline) for count data with replicates (especially for small sample sizes). For data without replicates, the DEGES-based pipeline with DESeq2 (called *SSS-S*) can be recommended.

**Electronic supplementary material:**

The online version of this article (doi:10.1186/s12859-015-0794-7) contains supplementary material, which is available to authorized users.

## Background

RNA sequencing (RNA-seq) is a basic tool for measuring expressions of multiple genomic loci [1–5]. One important goal for RNA-seq is to identify of differentially expressed genes (DEGs) under different conditions. Researchers typically start the differential expression (DE) analysis with a so-called “count matrix”, where each row indicates the gene (or exons or genomic loci), each column indicates the sample, and each cell indicates the number of reads mapped to the gene in the sample [5–9]. There are roughly four levels of resolution in current DE analysis: gene-, transcript-, exon-, and base-level. Examples of the DE methods for individual levels are (i) edgeR [10], DESeq [11], and TCC [12] for gene-level; (ii) Cuffdiff2 [13], IUTA [14], and SplicingCompass [15] for transcript-level; (iii) DEXSeq [16] and NPEBseq [17] for exon-level; and (iv) DER Finder [18] for base-level. Many methods can perform DE analysis for multiple levels (e.g., Cuffdiff2 can perform both gene- and transcript-level analysis) and are provided as R/Bioconductor packages [19, 20].

Read counts across technical replicates derived from a single source fit to a Poisson distribution [3, 21]. For data on biological replicates (BRs) derived from different individuals, the gene-level counts well fit to an over-dispersed Poisson distribution such as a negative-binomial (NB) model [10, 11, 22], beta-binomial (BB) model [5, 23], Poisson-Tweedie model [6], and so on. In particular, the Poisson-Tweedie model well captures the biological variation (especially for zero-inflation and heavy tail behavior, for details see [6]) when many BRs are available. As an increase in sample size (i.e., the number of replicate samples) precedes an increase in sequencing depth (i.e., the number of sequenced reads) [24–26], a more complex model such as Poisson-Tweedie may be the first choice for count data with many BRs. However, as many replicates are still difficult to take due to sequencing cost and the small amount of the target RNA sample, RNA-seq data with few BRs have mainly been stored. Two R packages based on the NB model (edgeR and DESeq) have been widely used as a common choice for DE analysis of RNA-seq data with few BRs [9–11, 27].

In general, the DE analysis consists of two steps (data normalization *X* and DEG identification *Y*), and each R package has its own methods for the *X*-*Y* pipeline [12]. The aim of normalization is to make the normalized counts for non-DEGs similar between all samples [28]. The edgeR and DESeq manipulate the raw count data as input. They first calculate normalization factors (or size factors) for individual samples as *X*, then construct the model (i.e., estimate the parameters on the model in which the calculated normalization factors are used to re-scale the raw counts), and calculate *p*-values (i.e., perform the statistical test using the model) as *Y*. Previous studies have demonstrated that *X* has more impact than *Y* on the ranked gene list [8, 29, 30] and that two normalization methods implemented in the two packages (edgeR and DESeq) generally give satisfactory results [31]. While the normalization method provided in edgeR is termed TMM (trimmed mean of M-values) [32], we here call the default pipelines *X*-*Y* for edgeR and DESeq “edgeR-edgeR (or *E-E*)” and “DESeq-DESeq (or *D-D*)”, respectively.

We previously proposed a multi-step normalization procedure called TbT [8]. TbT consists of three steps: *X* using TMM (step 1), *Y* using an empirical Bayesian method implemented in the baySeq package [22] (step 2), and *X* using TMM after elimination of the estimated DEGs (step 3) comprising the TMM-baySeq-TMM normalization pipeline. The key concept is to alleviate the negative effect of potential DEGs before calculating the normalization factors in step 3. As mentioned previously [8], the DEG elimination strategy (called DEGES) can be repeated until the calculated normalization factors converge. The iterative TbT can be described as a TMM-(baySeq-TMM)_*n*_ procedure. Accordingly, a generalized pipeline with the multi-step normalization can be described as *X*-(*Y*-*X*)_*n*_-*Y* in which the *X*-(*Y*-*X*)_*n*_ with *n* > = 2 corresponds to the iterative DEGES-based normalization.

Our TCC package [12] implements the proposed pipeline *X*-(*Y*-*X*)_*n*_-*Y*. Recommendations are made from an extensive simulation analysis: (i) edgeR-(edgeR-edgeR)_3_-edgeR on two-group RNA-seq data with few replicates and (ii) DESeq-(DESeq-DESeq)_3_-DESeq on two-group data without replicates [12]. However, similar to many other studies [24–28, 33], the performance evaluations were limited to a two-group comparison. While many R packages as well as TCC can perform DE analysis on more complex experimental designs [5, 9, 22, 34–37], there have been few evaluation studies on RNA-seq data with those designs, e.g., multi-group data. The current study aims to evaluate 12 pipelines available in nine R packages when analyzing multi-group RNA-seq count data. Specifically, our primary interest is to investigate the effectiveness of the DEGES-based pipeline in TCC under such more complex designs. We report pipelines suitable for multi-group comparison.

## Results and discussion

To investigate the performance of DE pipelines for a multi-group comparison, a total of 12 pipelines available in the nine packages were mainly evaluated in this study: TCC (ver. 1.7.15) [12], edgeR (ver. 3.8.5) [10], DESeq (ver. 1.18.0) [11], DESeq2 (ver. 1.6.3) [35], voom [38] in limma (ver. 3.22.1) [39], SAMseq [40] in samr (ver. 2.0), PoissonSeq (ver. 1.1.2) [41], baySeq (ver. 2.0.50) [22], and EBSeq (ver. 1.6.0) [42]. Note that TCC can perform several combinations for the DE pipeline *X*-(*Y*-*X*)_*n*_-*Y* with *n* = 3 as recommended [12]. We sometimes refer to this DEGES-based pipeline as *XYX*-*Y* with the fixed number of *n* for short. We basically confine individual methods (*X* and *Y*) in each pipeline to functions provided by the same packages (i.e., edgeR or DESeq2) for simplicity. For example, the edgeR-related pipeline is “edgeR-(edgeR-edgeR)_3_-edgeR”, where *X* = TMM and *Y* = the DEG identification method, implemented in edgeR. Although we previously termed this pipeline “iDEGES/edgeR-edgeR” [12], here we abbreviate it to *EEE-E* for convenience. Similarly, the “DESeq-(DESeq-DESeq)_3_-DESeq” pipeline can be shortened to *DDD-D*. This is because (1) users can select, for example, different DEG identification methods *Y* for steps 2 and 4 and (2) we will discuss some possible combinations such as *DED-S* for the “DESeq-(edgeR-DESeq)_3_-DESeq2” pipeline. In this sense, the DEGES-based pipeline can also be denoted as *X*-(*Y*-*X*)_*n*_-*Z* or *XYX*-*Z*.

Following our previous studies [8, 12], we here demonstrate the performance of these pipelines mainly on the basis of same evaluation metric and simulation framework. We use the area under the ROC curve (AUC) as a main measure of comparison, which evaluates both sensitivity and specificity of the pipelines simultaneously [28, 43–48]. To perform the multi-group comparison as simply as possible, we focus here on the three-group data (i.e., G1 vs. G2 vs. G3) with equal numbers of replicates (i.e., 1, 3, 6, and 9 replicates per group; Nrep = 1, 3, 6, and 9). The gene ranking was performed on the basis of an ANOVA-like *p*-value or the derivatives, where a low *p*-value for a gene indicates a high degree of DE in at least one of the groups compared. The simulation conditions are as follows: the total number of genes is 10,000 (Ngene = 10000), 5 or 25 % of the genes are DEGs (P_DEG_ = 5 or 25 %), the levels of DE are four-fold in individual groups, and the proportions of DEGs up-regulated in individual groups (P_G1_, P_G2_, P_G3_) are (1/3, 1/3, 1/3), (0.5, 0.3, 0.2), (0.5, 0.4, 0.1), (0.6, 0.2, 0.2), (0.6, 0.3, 0.1), (0.7, 0.2, 0.1), and (0.8, 0.1, 0.1).

### Simulation data with replicates

We first assessed the performances of a total of 12 pipelines: three pipelines in TCC (i.e., *EEE-E*, *DDD-D*, and *SSS-S*), edgeR, edgeR_robust, DESeq, DESeq2, voom, SAMseq, PoissonSeq, baySeq, and EBSeq. Table 1 lists the average AUC values of 100 trials between the ranked gene lists and the truth for various simulation conditions with Nrep = 3. Overall, the AUC values for the *EEE-E* pipeline were the highest and similar across the seven different proportions of DEGs up-regulated in individual groups (P_G1_, P_G2_, P_G3_). The edgeR (i.e., the pipeline *E-E*) performed the second best overall. *EEE-E* and edgeR performed comparably under the unbiased proportion of DEGs in individual groups (1/3, 1/3, 1/3). This is quite reasonable because the *EEE-E* can be viewed as an iterative edgeR pipeline and their theoretical performances are the same under the unbiased condition [12]. Similar to the relationship between *EEE-E* and edgeR, the *DDD-D* (or *SSS-S*) can be viewed as an iterative DESeq (or DESeq2) pipeline. As expected, *DDD-D* (or *SSS-S*) consistently outperformed DESeq (or DESeq2) in all simulation conditions except for the unbiased situations.

**Table 1:** Average AUC values for simulation data with replicates

PG1	33 %	50 %	50 %	60 %	60 %	70 %	80 %
PG2	33 %	30 %	40 %	20 %	30 %	20 %	10 %
PG3	33 %	20 %	10 %	20 %	10 %	10 %	10 %
(a) P_DEG_ = 5 %							
*EEE-E* (TCC)	91.57	**91.50**	**91.50**	**91.43**	**91.42**	**91.45**	**91.46**
*DDD-D* (TCC)	90.70	90.62	90.64	90.54	90.55	90.59	90.62
*SSS-S* (TCC)	88.34	88.33	88.30	88.24	88.23	88.21	88.30
*E-E* (edgeR)	**91.58**	91.48	91.47	91.38	91.37	91.38	91.34
edgeR_robust	90.95	90.86	90.85	90.75	90.74	90.74	90.73
*D-D* (DESeq)	90.71	90.60	90.60	90.50	90.49	90.50	90.48
*S-S* (DESeq2)	88.34	88.31	88.26	88.19	88.17	88.11	88.14
voom	87.16	87.01	86.99	86.88	86.91	86.88	86.86
SAMseq	85.04	84.97	84.93	84.83	84.88	84.88	84.91
PoissonSeq	87.31	87.25	87.25	87.19	87.17	87.22	87.23
baySeq	90.24	90.21	90.21	90.22	90.17	90.13	90.07
EBSeq	85.77	85.85	85.78	85.81	85.73	85.71	85.77
(b) P_DEG_ = 25 %							
*EEE-E* (TCC)	91.47	**91.46**	**91.45**	**91.45**	**91.43**	**91.42**	**91.37**
*DDD-D* (TCC)	90.77	90.73	90.72	90.70	90.68	90.65	90.57
*SSS-S* (TCC)	88.13	88.11	88.13	88.14	88.12	88.09	88.06
*E-E* (edgeR)	**91.47**	91.30	91.18	91.06	90.98	90.62	89.97
edgeR_robust	90.89	90.69	90.57	90.43	90.34	89.97	89.27
*D-D* (DESeq)	90.77	90.54	90.37	90.25	90.15	89.73	89.04
*S-S* (DESeq2)	88.12	87.83	87.62	87.49	87.36	86.79	85.92
voom	87.08	86.71	86.52	86.29	86.18	85.60	84.56
SAMseq	84.95	84.82	84.82	84.77	84.75	84.72	84.63
PoissonSeq	87.22	87.18	87.14	87.13	87.11	87.06	86.97
baySeq	90.34	90.13	90.07	89.92	89.83	89.52	88.86
EBSeq	85.82	85.61	85.49	85.34	85.30	84.74	84.02

Average AUC values (%) of 100 trials for each simulation condition are shown: (a) P_DEG_ = 5 % and (b) P_DEG_ = 25 %. Simulation data contain a total of 10,000 genes: P_DEG_ % of genes is for DEGs, P_G1_ % of P_DEG_ in G1 is higher than in the other groups, and each group has three BRs (Nrep = 3). Seven conditions are shown in total. The highest AUC value for each condition is in bold

We observed similar AUC values across the seven different proportions of DEGs for individual pipelines at P_DEG_ = 5 % (Table 1a). When a higher amount of DEGs was introduced (i.e., P_DEG_ = 25 %; Table 1b), the performances generally worsened as the degrees of biases increased (i.e., from left to right in Table 1). For example, the AUC values for voom under the unbiased (1/3, 1/3, 1/3) and most biased (0.8, 0.1, 0.1) proportions decreased from 87.08 to 84.56 %. We observed relatively poor performances for EBSeq and voom. This is consistent with a previous simulation study on two-group data with a low number of BRs (Nrep = 2) [28]. A possible explanation of these results is that EBSeq was originally developed to detect DE isoforms (not DEGs) [41] and the large body of methodology in voom is for microarray data (not RNA-seq count data) [38]. Our current evaluation focuses on the gene-level RNA-seq count data and does not address the problem of such a detailed resolution of DE analysis. SAMseq and PoissonSeq performed stably across different proportions. This is probably because both methods are non-parametric ones that do not assume any particular distribution for the data and are generally robust against such biased situations. These methods, however, performed poorly overall. Additional file 1 is the R code for obtaining these results.

It should be noted that the relative performances for EBSeq tend to improve as the number of replicates per group increases (Nrep = 6 and 9; see Sheet 2 and 3 in Additional file 2). In particular, EBSeq consistently outperformed the others when Nrep = 9 and P_DEG_ = 5 %, suggesting that the DEGES-based pipeline based on EBSeq could produce a more accurate ranked gene list. However, as previously discussed for the DEGES-based pipeline based on baySeq [12], Bayesian methods (EBSeq and baySeq) generally require huge computation time (see Sheet 4 in Additional file 2). While the computation can be parallelized, the implementation of DEGES for EBSeq might be unfeasible.

Recall that the level of DE for DEGs was four-fold in this simulation framework and the shape of the distribution for introduced DEGs is the same as that of non-DEGs [8]. Although the simulation framework has been used [8; 12; 32], this may weaken the validity of the current simulation framework. To mitigate this concern, we performed simulations with different distributions of DE when introducing DEGs. In this simulation, the fold-changes for DEGs were randomly sampled from “1.2 + a gamma distribution with shape = 2.0 and scale = 0.5”, giving mean fold-change of 2.2 (=1.2 + 2.0 × 0.5). Similar to the results with a fixed level of DE (four-fold for all DEGs), *EEE-E* performed the best overall (see Sheet 5 in Additional file 2). While a more extensive study with other simulation settings should still be performed, this trend suggests that different distributions of DE does not have much impact on the DE results. The functionality for generating the different distributions of DE in the “simulateReadCounts” function will be available in TCC ver. 1.9.3 or higher.

As mentioned above, TCC can perform various combinations for the DEGES-based DE pipeline *X*-(*Y*-*X*)_*n*_-*Z* or *XYX*-*Z*, where *Y* and *Z* are the DEG identification methods and *X* is the normalization method. We investigated the effect of the individual methods (used as *X*, *Y*, and *Z*) by analyzing a total of 12 pipelines (eight DEGES-based pipelines and four non-DEGES-based pipelines). Table 2 shows the average AUC values for these pipelines. Note that the values in Tables 1 and 2 are comparable and that those for the four pipelines (*EEE-E*, *DDD-D*, *E-E*, and *D-D*; colored gray in Table 2) are provided in both tables. It is clear that choosing *Z* has more impact on the gene ranking accuracy than choosing *Y* and that using the DEG identification method provided in edgeR in both *Y* and *Z* can be recommended. In comparison with the two normalization methods in *X* in the eight DEGES-based pipelines, the method in DESeq (denoted as “*D*”) gave slightly higher AUC values than the TMM normalization method in edgeR (denoted as “*E*”). However, the superiority of DESeq in *X* was not observed when the four non-DEGES-based pipelines *X*-*Z* were compared, where edgeR (i.e., the TMM normalization method) outperformed DESeq. In any case, the different choices in *X* have less impact than those in *Y* and *Z*. Additional file 3 is the R code for obtaining the results shown in Table 2.

**Table 2:** Effect of different choices for the possible pipelines in TCC

PG1	33 %	50 %	50 %	60 %	60 %	70 %	80 %
PG2	33 %	30 %	40 %	20 %	30 %	20 %	10 %
PG3	33 %	20 %	10 %	20 %	10 %	10 %	10 %
(a) P_DEG_ = 5 %							
***EEE-E***	91.57	91.50	91.50	**91.43**	91.42	91.45	91.46
*DED-E*	91.57	**91.50**	**91.50**	91.43	**91.42**	**91.46**	**91.47**
*EDE-E*	91.57	91.50	91.50	91.43	91.42	91.45	91.46
*DDD-E*	91.57	91.50	91.50	91.43	91.42	91.45	91.46
*EEE-D*	90.70	90.62	90.64	90.54	90.55	90.58	90.62
*DED-D*	90.71	90.62	90.64	90.54	90.55	90.59	90.62
*EDE-D*	90.70	90.62	90.64	90.54	90.55	90.58	90.62
***DDD-D***	90.70	90.62	90.64	90.54	90.55	90.59	90.62
***E-E*** (edgeR)	91.58	91.48	91.47	91.38	91.37	91.38	91.34
*D-E*	**91.58**	91.48	91.46	91.38	91.36	91.36	91.32
*E-D*	90.70	90.61	90.61	90.50	90.50	90.51	90.50
***D-D*** (DESeq)	90.71	90.60	90.60	90.50	90.49	90.50	90.48
(b) P_DEG_ = 25 %							
***EEE-E***	91.47	91.46	91.45	91.45	91.43	91.42	91.37
*DED-E*	91.47	**91.46**	**91.47**	**91.47**	**91.45**	**91.45**	**91.43**
*EDE-E*	91.47	91.43	91.41	91.40	91.36	91.30	91.19
*DDD-E*	91.47	91.44	91.43	91.42	91.39	91.36	91.29
*EEE-D*	90.77	90.74	90.74	90.73	90.71	90.71	90.65
*DED-D*	90.77	90.74	90.76	90.75	90.73	90.74	90.71
*EDE-D*	90.77	90.71	90.70	90.68	90.64	90.60	90.47
***DDD-D***	90.77	90.73	90.72	90.70	90.68	90.65	90.57
***E-E*** (edgeR)	91.47	91.30	91.18	91.06	90.98	90.62	89.97
*D-E*	**91.48**	91.25	91.08	90.96	90.86	90.44	89.75
*E-D*	90.77	90.59	90.48	90.35	90.26	89.92	89.25
***D-D*** (DESeq)	90.77	90.54	90.37	90.25	90.15	89.73	89.04

Legends are basically the same as in Table 1. Results of a total of 12 pipelines are shown. The AUC values for four pipelines (*EEE-E*, *DDD-D*, *E-E*, and *D-D*) in bold are also shown in Table 1. The *DED-E* pipeline outperforms the others overall

Surprisingly, the best pipeline was *DED-E*, followed by *EEE-E* and *DDD-E* (Table 2b). The *DED-E* and *DDD-E* pipelines consist of methods provided by different packages. For example, *DED-E*, the “DESeq-(edgeR-DESeq)_3_-edgeR” pipeline, consists of the normalization method in DESeq as *X* and the DEG identification method in edgeR as *Y* and *Z*. These results suggest that in some cases, the suitable choices of the best pipeline may slightly improve DE results. We should note that the current simulation data are generated by the “simulateReadCounts” function in TCC. This is simply because, to the best of our knowledge, TCC only provides the R function that can generate multi-group simulation count data. TCC simulates all counts using NB distributions, implying that this simulation framework advantageously acts on the classical R packages such as edgeR and DESeq. This is probably the main reason for poor performances of two recently published packages (edgeR_robust and DESeq2; see Table 1); those are the advanced versions for edgeR and DESeq, respectively, and are robust against count outliers such as abnormally high counts (for details, see [35, 36]). To the best of our knowledge, only one R package, compcodeR [48], can generate simulation count data with outliers, but it has been restricted to only two-group comparisons so far. Extending the simulation framework of compcodeR to multi-group data may allow different pipelines to be compared more equally.

### Simulation data without replicates

Unlike (multi-group) count data with replicates, there are few packages that can manipulate count data without replicates. These include TCC, edgeR, DESeq, DESeq2, and so on. We here evaluated a total of 20 pipelines (13 DEGES-based pipelines and seven non-DEGES-based pipelines). Table 3 shows the results for simulation data without replicates under P_DEG_ = 25 %. When three original non-DEGES-based pipelines *X*-*Z* are compared, DESeq2 (i.e., *S-S*) performed the best, followed by DESeq (*D-D*) and edgeR (*E-E*). This is completely different from the results in Table 2. When 13 DEGES-based pipelines *XYX*-*Z* are compared, choosing *Z* for the DEGES-based pipeline has more impact on the gene ranking accuracy than choosing *Y* (similar to Table 2) and that using the DEG identification method provided in DESeq2 (i.e., *S*) can be recommended as *Z*. This result may possibly be explained by the removal of outliers that do not fit the distributional assumptions of the model [40]: DESeq2 [35] implements a functionality for detecting and removing outliers on the basis of Cook’s distance [49]. In the situation of count data without replicates, DEGs tend to be flagged as outliers: Cook’s distances are generally greater for DEGs than for non-DEGs. The negative effect of 25 % DEGs introduced in this simulation framework could therefore be weakened.

**Table 3:** – Average AUC values for simulation data without replicates

PG1	33 %	50 %	50 %	60 %	60 %	70 %	80 %
PG2	33 %	30 %	40 %	20 %	30 %	20 %	10 %
PG3	33 %	20 %	10 %	20 %	10 %	10 %	10 %
*EEE-E*	77.15	76.88	76.78	76.63	76.88	76.15	75.48
*DED-E*	77.15	76.86	76.73	76.59	76.86	76.08	75.41
*EDE-E*	77.15	76.88	76.79	76.64	76.88	76.19	75.57
*DDD-E*	77.15	76.87	76.75	76.61	76.87	76.13	75.50
*EEE-D*	81.51	81.14	81.28	80.93	81.14	80.51	79.97
*DED-D*	81.52	81.14	81.25	80.90	81.14	80.45	79.90
*EDE-D*	81.49	81.14	81.28	80.94	81.14	80.55	80.05
*DDD-D*	81.51	81.15	81.26	80.91	81.15	80.49	79.98
*E-E* (edgeR)	77.15	76.87	76.76	76.60	76.87	76.10	75.36
*D-E*	77.15	76.86	76.71	76.57	76.86	76.04	75.35
*E-D*	81.49	81.13	81.27	80.91	81.13	80.46	79.86
*D-D* (DESeq)	81.53	81.12	81.23	80.88	81.12	80.41	79.84
*SSS-S*	82.46	82.18	82.08	81.98	82.18	81.52	80.97
*EEE-S*	**82.46**	82.18	82.08	81.98	82.18	81.50	80.89
*DED-S*	82.46	82.17	82.04	81.95	82.17	81.43	80.81
*EDE-S*	82.46	**82.18**	**82.09**	**82.00**	**82.18**	**81.54**	**80.97**
*DDD-S*	82.46	82.17	82.06	81.97	82.17	81.48	80.90
*S-S* (DESeq2)	82.46	82.16	82.01	81.92	82.16	81.38	80.73
*E-S*	82.46	82.17	82.07	81.96	82.17	81.45	80.76
*D-S*	82.46	82.16	82.02	81.93	82.16	81.39	80.74

Legends are basically the same as in Table 1. Results of a total of 20 pipelines under P_DEG_ = 25 % are shown. The *EDE-S* pipeline outperforms the others overall

In addition to the model construction only with non-outliers in the Z step, the DEGES-based normalization in the *XYX* step also slightly but reliably improves ranked gene lists. For example, the AUC values higher for *SSS-S* than *S-S* (i.e., DESeq2) are by virtue of the multi-step normalization strategy originally proposed by Kadota et al. [8]. However, as also discussed in the TCC paper [12], DESeq and DESeq2 generally estimate false discovery rates (FDR) more conservatively than others [9]. Indeed, we observed that the numbers of potential DEGs satisfying 10 % FDR in step 2 (i.e., the *Y* step) in the *SSS-S* pipeline were nearly zero (i.e., the estimated P_DEG_ values were 0 %) in all simulations, although the actual P_DEG_ values were 25 %. This is reasonable because any attempt to work without replicates will lead to conclusions of very limited reliability [12]. TCC employs a predefined floor P_DEG_ value (=5 %) to obtain certain differences between the DEGES-based approach *SSS-S* and non-DEGES-based approach *S-S*: at least 5 % of the top-ranked genes are not used when the normalization factors are calculated at step 3 in the *XYX* pipeline. As an estimated P_DEG_ value of *x*% tends to work better when simulation data with the same P_DEG_ value are analyzed, accurate estimation is the next important task. Additional file 4 is the R code for obtaining the results shown in Table 3.

### Real data with replicates

In addition to the simulation study, we also analyzed a real RNA-seq count dataset sequenced from the three species (i.e., the three-group data): humans (HS), chimpanzees (PT), and rhesus macaques (RM) [50]. Briefly, Blekhman et al. studied expression levels of liver samples from three males (M1, M2, and M3) and three females (F1, F2, and F3) from each species, giving a total of six different individuals (i.e., six biological replicates) for each species. Since they performed duplicate experiments for each individual (i.e., two technical replicates), the publicly available raw count matrix consists of 20,689 genes × 36 samples (=3 species × 2 sexes × 3 biological replicates × 2 technical replicates). To correctly estimate the biological variation and make the assumed structure of input data, we summed and collapsed the count data of technical replicates, giving a reduced number of columns in the count matrix (i.e., 18 samples; three species × 2 sexes × 3 biological replicates). We here compared a total of 12 pipelines in light of the overall similarity of ranked gene lists, the number of shared DEGs satisfying an FDR threshold, and so on. To compare these pipelines as simply as possible, we regarded this dataset as a single-factor experimental design of three species where each has six biological replicates (i.e., HS_rep1-6 vs. PT_rep1-6 vs. RM_rep1-6). The full R code for analyzing this dataset is provided in Additional file 5. The results of sample clustering applied to these raw and collapsed count datasets are given in Additional file 6 (as part of the results in Additional file 5).

**Fig. 1:**
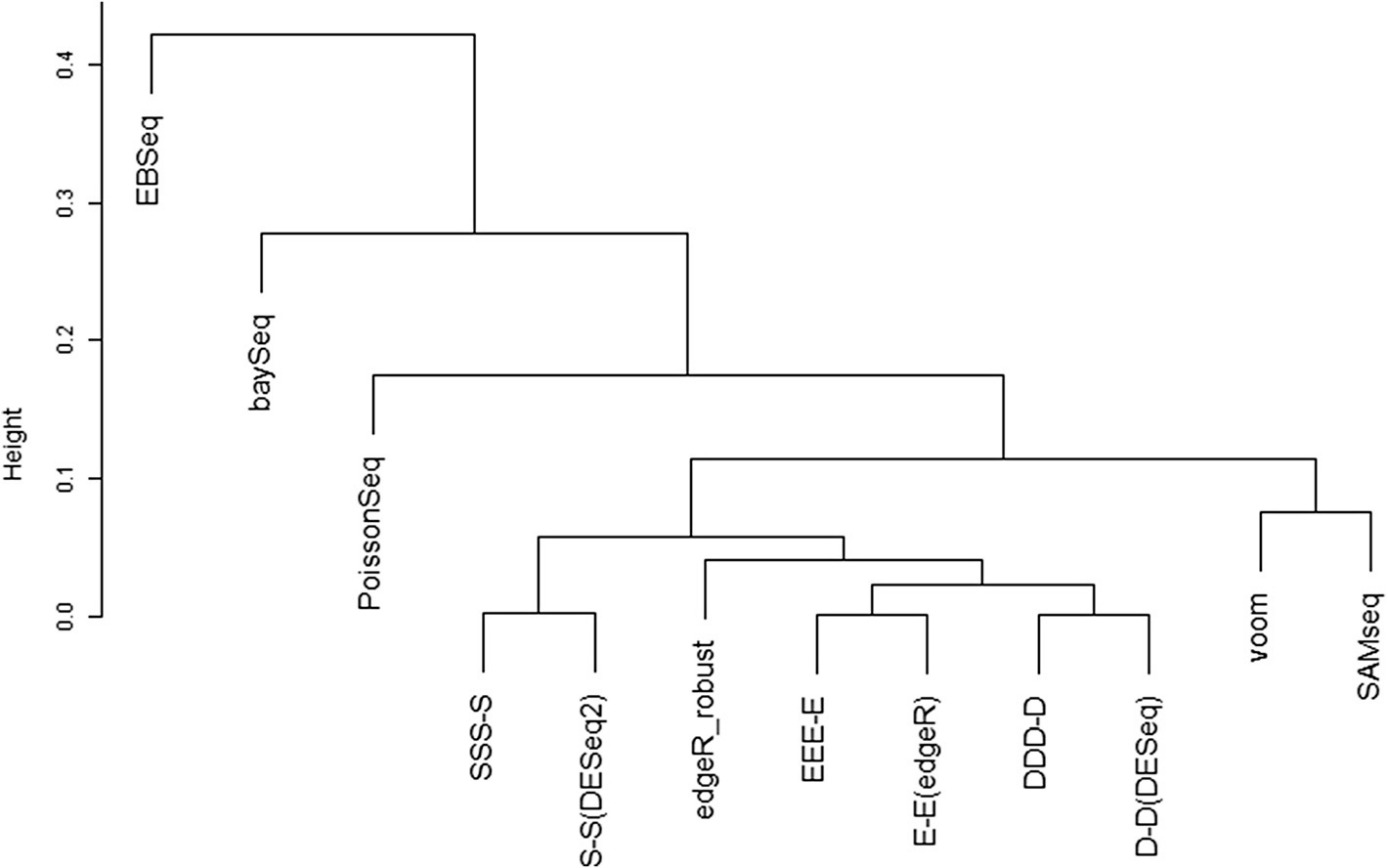
Overall similarity of 12 ranked gene lists applied for Blekhman’s real count data. The dendrogram of average-linkage clustering is shown. Spearman’s rank correlation coefficient (*r*) is used as a similarity metric; left-hand scale represents (1 - *r*)

Figure 1 shows the dendrogram of average-linkage clustering for the 12 ranked gene lists. Seven pipelines located in the center (from *SSS-S* to *D-D*) show similar ranked gene lists. This is mainly because the seven pipelines from the four packages (TCC, edgeR, DESeq, and DESeq2) commonly employ a generalized linear model (GLM) framework. Indeed, the minimum value of Spearman’s correlation coefficients (*r*) among the seven pipelines was 0.9240. It is also noteworthy that ranked gene lists produced from TCC’s iterative strategies and the corresponding original non-iterative strategies are particularly similar. For example, the *r* between *EEE-E* from TCC and *E-E* from edgeR was 0.9999, implying that these data are not extremely biased in light of the proportions of DEGs up- and/or down-regulated in individual groups (P_G1_, P_G2_, P_G3_). That is, the proportions of DEGs in these data (P_G1_, P_G2_, P_G3_) are rather closer to (1/3, 1/3, 1/3) than, for example, (0.8, 0.1, 0.1) or (0.0, 0.9, 0.1).

Note that the dendrogram shown in Fig. 1 does not necessarily indicate the superiority of the seven GLM-based pipelines over the others such as EBSeq and baySeq. For example, EBSeq employs an empirical Bayesian framework that returns the posterior probabilities for each of the five possible expression patterns (or models) to each gene. We here used the posterior probability obtained from the “non-DEG” pattern as a surrogate estimate for the adjusted *p*-values and ranked genes in ascending order of the values. This is probably the main reason for EBSeq having lower similarity than the others. We also confirmed this trend with some simulation data. As shown in Sheet 2 in Additional file 2, EBSeq had the highest average AUC values in the simulation condition: P_DEG_ = 5 %, (0.5, 0.4, 0.1) for (P_G1_, P_G2_, P_G3_), and Nrep = 9. A typical dendrogram of 12 ranked gene lists obtained from this simulation condition is given in Additional file 7. In this trial, while EBSeq and baySeq formed one of the two major clusters, those AUC values were not the top two: the ranks for EBSeq and baySeq were the 1st and 6th, respectively. These results indicate that the low similarities of ranked gene lists between Bayesian pipelines (such as EBSeq and baySeq) and the GLM-based pipelines do not matter.

We compared the numbers of DEGs obtained from individual pipelines and the overlaps between all pairs of pipelines (see Additional file 8). We found that different pipelines could produce considerably different numbers of DEGs. Indeed, the numbers widely ranged from 3832 (18.5 % of all genes; DESeq) to 9453 (45.7 %; SAMseq). This trend is consistent with that in a previous comparative study [28]. As expected from Fig. 1, we observed similar numbers of DEGs between the three DEGES-based pipelines (*EEE-E*, *DDD-D*, and *SSS-S*) and the corresponding non-DEGES-based ones (*E-E*, *D-D*, and *S-S*). The Jaccard coefficients, defined as “intersection/union” for two sets of DEGs, for the three pairs (*EEE-E* vs. *E-E*, *DDD-D* vs. *D-D*, and *SSS-S* vs. *S-S*) were top-ranked among a total of 66 possible pairs. For example, both *EEE-E* in TCC and *E-E* in edgeR reported the same numbers of DEGs (=7247). Of these, 7208 DEGs (99.46 %) were common, and the Jaccard coefficient was 7208 / 7286 = 0.9893 (see Additional file 8). The overall number of common genes across the 12 sets of DEGs was 2376. Since individual sets were identified under the 5 % FDR threshold, 95 % of the 2376 common DEGs can statistically be regarded as *confident*.

We next classified the expression patterns of the DEGs obtained from the 12 pipelines (Table 4). We here assigned individual DEGs to one of the ten possible patterns defined in baySeq [22]; this package returns one of these patterns to each gene. The *background* information for this data is shown in the “all_genes” row in Table 4. The “common” row indicates the percentages of individual expression patterns for the 2376 common DEGs. The remaining rows (from *EEE-E* to EBSeq) show the distributions for each of the pipelines. It is reasonable that no DEGs identified by individual pipelines are assigned as a flat expression pattern (i.e., G1 = G2 = G3) for the HS (G1) vs. PT (G2) vs. RM (G3) comparison. We found that most DEGs were assigned preferably to one of four patterns (G1 > G2 > G3, G2 > G1 > G3, G3 > G1 > G2, and G3 > G2 > G1) and unpreferably to one of two patterns (G1 > G3 > G2 and G2 > G3 > G1). That is, up- (or down-) regulation in G1 for DEGs tends to coincide with G2 more than G3. This can also be seen in the results from sample clustering of the raw count data (see Additional file 6), implying that we can roughly predict the DE results such as those shown in Table 4 from the overall similarities of samples on the raw count data.

**Table 4:** – Classification of expression patterns for DEGs

	G1 = G2 = G3	G1 > G2 = G3	G1 > G2 > G3	G1 > G3 > G2	G2 > G1 = G3	G2 > G1 > G3	G2 > G3 > G1	G3 > G1 = G2	G3 > G1 > G2	G3 > G2 > G1	Total
all_genes	13.5	2.2	15.1	8.7	2.3	15.9	9.4	2.9	15.1	14.8	20689
common	0.0	0.1	23.2	5.8	0.2	26.4	5.7	0.7	18.6	19.2	2376
*EEE-E*	0.0	0.6	20.7	7.4	0.7	21.9	8.1	1.6	19.9	19.2	7247
*DDD-D*	0.0	0.4	25.0	7.3	0.6	25.0	6.0	1.4	17.3	17.1	3850
*SSS-S*	0.0	0.2	19.3	7.1	0.3	21.7	9.4	0.9	19.9	21.2	7295
*E-E* (edgeR)	0.0	0.6	20.4	7.3	0.7	22.1	8.3	1.6	19.7	19.3	7247
edgeR_robust	0.0	0.3	20.6	8.4	0.5	22.0	8.8	1.2	19.1	18.9	8076
*D-D* (DESeq)	0.0	0.4	24.3	7.2	0.6	24.2	6.0	1.4	17.8	18.1	3832
*S-S* (DESeq2)	0.0	0.2	20.4	8.0	0.3	21.8	8.9	0.8	19.7	19.9	7585
voom	0.0	0.7	21.3	7.7	0.7	22.5	8.2	1.3	18.7	19.0	7016
SAMseq	0.0	0.2	20.9	9.7	0.3	21.8	9.2	0.8	18.9	18.3	9453
PoissonSeq	0.0	0.0	19.5	8.9	0.1	22.2	9.4	0.3	20.3	19.3	6613
baySeq	0.0	0.8	21.0	5.5	1.3	23.7	6.3	2.8	19.0	19.6	3975
EBSeq	0.0	0.0	21.0	7.0	0.1	23.7	7.1	0.3	20.8	19.9	5699

Percentages of genes assigned to each of the ten possible patterns defined as baySeq. Numbers in the “Total” column indicate the numbers of genes. For example, baySeq assigned 13.5 % of 20,689 genes as “G1 = G2 = G3.”

When comparing the distributions of patterns for DEGs between pipelines, we saw high similarities overall. If anything, baySeq showed a distribution relatively different from the others in light of the higher percentages for three patterns (G1 > G2 = G3, G2 > G1 = G3, and G3 > G1 = G2). This kind of classification can also be performed using EBSeq [42]. EBSeq defines a total of five possible patterns when comparing three groups: Pattern 1 for non-DEG (i.e., G1 = G2 = G3), Pattern 2 for differential expression (DE) in G3 (G1 = G2 < G3 and G1 = G2 > G3), Pattern 3 for DE in G2 (G2 > G1 = G3 and G2 < G1 = G3), Pattern 4 for DE in G1 (G1 > G2 = G3 and G1 < G2 = G3), and Pattern 5 for DE among all groups. Similar to baySeq, EBSeq also returns one of these patterns to each gene. The results of classification based on EBSeq are given in Additional file 9. Similar to the results from baySeq (Table 4), we observed that nearly half the DEGs were assigned to Pattern 2, where the expression patterns between G1 and G2 tend to be more similar than for G3. We also observed that the distribution for baySeq is relatively different from the others, e.g., lower percentages in Patterns 3 and 4 and a higher percentage in Pattern 5.

We finally assessed the reproducibility of ranked gene lists. Remember that the real dataset we analyzed here consists of three groups, each of which has six BRs (we denote this dataset as “rep1-6”). In addition to the original three-group comparison with six replicates (i.e., HS_rep1-6 vs. PT_rep1-6 vs. RM_rep1-6), we also performed three three-group comparisons by dividing the original dataset into three; individual subsets consist of two BRs for each group. For example, the first subset (say “rep1-2”) consists of a total of six samples for comparing HS_rep1-2, PT_rep1-2, and RM_rep1-2. Likewise, the third subset (“rep5-6”) is for comparing “HS_rep5-6 vs. PT_rep5-6 vs. RM_rep5-6”. After performing the DE analysis for the three subsets (i.e., rep1-2, rep3-4, and rep5-6), we obtained three ranked gene lists for these subsets. Accordingly, there are a total of four ranked gene lists (rep1-2, rep3-4, rep5-6, and rep1-6) for each pipeline. We evaluated the reproducibility of ranked gene lists (i) for each subset to the original dataset (i.e., rep1-6 vs. rep1-2, rep1-6 vs. rep3-4, and rep1-6 vs. rep5-6) and (ii) among the three subsets (i.e., rep1-2 vs. rep3-4 vs. rep5-6).

**Fig. 2:**
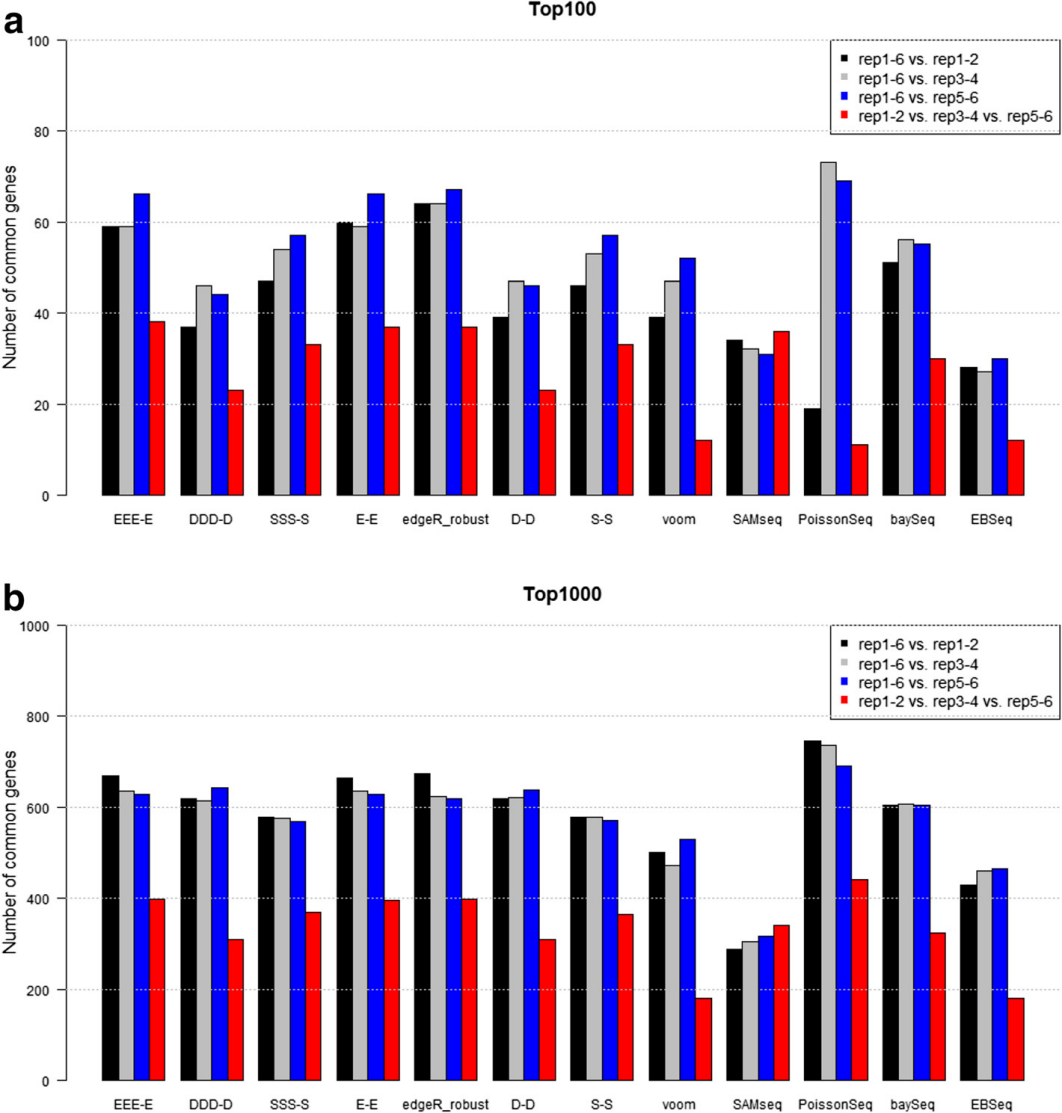
Reproducibility between ranked gene lists. Numbers of common genes between top-ranked genes for individual pipelines are shown: (**a**) results for 100 top-ranked gene lists and (**b**) results for 1000 top-ranked gene lists. Bars in black (rep1-6 vs. rep1-2), gray (rep1-6 vs. rep3-4), and blue (rep1-6 vs. rep5-6) in Fig. 2a indicate the numbers of common genes between the two sets of 100 top-ranked genes obtained from the individual pipelines. For example, the gray bar (rep1-6 vs. rep3-4) for *DDD-D* in Fig. 2a indicates that there were 46 common genes when the 100 top-ranked genes from the dataset rep1-6 are compared with the 100 top-ranked genes from the dataset rep3-4. Analogously, bars in red (rep1-2 vs. rep3-4 vs. rep5-6) in Fig. 2b indicate the numbers of common genes between the three sets of 1000 top-ranked genes for the three datasets (rep1-2, rep3-4, and rep5-6). For example, the red bar for *EEE-E* in Fig. 2b indicates that there were 397 common genes (39.7 % of overlapping genes) when the three sets of gene lists (each of which contains 1000 top-ranked genes) obtained from the pipeline *EEE-E* for the three datasets were compared. The full R code for this analysis is given in Additional file 5

Figure 2 shows the numbers of common genes between the compared sets of top-ranked genes for individual pipelines: (a) for the top 100 and (b) for the top 1000. For example, there were 66 common genes when comparing the two sets (rep1-6 and rep5-6) of the 100 top-ranked genes obtained from the *EEE-E* pipeline (see the leftmost blue bar in Fig. 2a). As shown in Table 1 and Additional file 2, the more BRs we use, the more accurate the ranked gene lists we can obtain. Accordingly, the evaluation based on the reproducibility of ranked gene lists is analogous to a performance comparison when the available count data have only two BRs. Overall, we see high reproducibility for three edgeR-related pipelines (*EEE-E*, *E-E*, and edgeR_robust) and low reproducibility for two pipelines (SAMseq and EBSeq). This trend is consistent with the simulation results shown in Table 1 (i.e., three-group data with three BRs) and previous simulation results for two-group data with two BRs [28]. Although PoissonSeq showed the highest reproducibility when the 1000 top-ranked genes were evaluated (Fig. 2b), the performance seems unstable, especially on < 200 top-ranked genes. This is mainly due to low reproducibility of the ranked gene list for rep1-2 to the list for rep1-6. Although we saw a plausible outlying sample (RMM2 or RM_rep5) in the dendrogram of sample clustering for the raw count data, it would not have been related to the dissimilarity of ranked gene lists between rep1-2 and rep1-6. The percentages of overlapping/common genes (POGs) for any numbers of top-ranked genes are given in Additional file 10.

### Effect of different choices for options

In general, there are multiple options for some functions, and different choices may result in different ranked gene lists. We investigated the effect of different choices for two representative pipelines (*E-E* and *D-D*) under one simulation condition (0.5, 0.4, 0.1) for (P_G1_, P_G2_, P_G3_) shown in Table 1a. For *E-E*, we evaluated a total of eight combinations, 4 *method* options (*“TMM”*, *“RLE”*, *“upperquartile”*, and *“none”*) in “calcNormFactors” function × 2 *test* options (*“chisq”* and *“F”*) in the “glmLRT” function, provided in edgeR. We observed quite similar performances between the two *test* options (*“chisq”* and *“F”*). The average AUC value when using the *method = ”TMM”* option was the highest (91.47 %), followed by *“RLE”* (91.46 %), *“upperquartile”* (91.40 %), and *“none”* (91.19 %). Since the best practice (i.e., using *method = “TMM”* and *test = “chisq”*) is the default in *E-E* (i.e., edgeR), the choices should be left unchanged. Currently, TCC does not allow these options to be changed when performing *EEE-E* that can be recommended for multi-group data with replicates.

We should note that *D-D* does not follow the simple conclusion described above (i.e., the default is the best). We evaluated a total of 18 combinations, 3 *method* options (*“pooled”*, *“pooled-CR”*, and *“blind”*) × 3 *sharingMode* options (*“maximum”*, *“fit-only”*, and *“gene-est-only”*) × 2 *fitType* options (*“parametric”* and *“local”*), in “estimateDispersions” function provided in DESeq. While the average AUC value for the suggested combination in DESeq (i.e., *method = “pooled”*, *sharingMode = “maximum”*, and *fitType = “parametric”*) was 90.60 %, the highest value in the 18 interrogated combinations was 91.69 %. Surprisingly, the best performing combination (i.e., *method = “blind”*, *sharingMode = “fit-only”*, and *fitType = “local”*) did not include any suggested choice.

We found that using both *method = “blind”* and *sharingMode = “fit-only”* was especially important to obtain high AUC value when analyzing count data with replicates. Recall that the combination was the default (or suggested) choices for DESeq when analyzing count data without replicates. The high AUC values with the *sharingMode = “fit-only”* option can be explained by the nature of simulation data (see the ‘Simulation data with replicates’ subsection). In other words, (i) the *sharingMode = “fit-only”* option is advantageous when existence of count outliers is not assumed (see the “estimateDispersions” function manual in DESeq) and (ii) the simulation data generated by the “simulateReadCounts” function in TCC do not have count outliers. The *method = “blind”* option ignores the group labels (G1 or G2 or G3) and can compute a gene’s empirical dispersion value even if there is no BRs. As described in the manual, this method can lead to loss of power (i.e., low sensitivity). The use of *method = “blind”* for count data with replicates cannot practically be recommended in light of potential low sensitivity. Most importantly, DESeq (i.e., *D-D*) is no longer recommended by the authors and DESeq2 (i.e., *S-S*) is recommended instead regardless of the number of BRs [35].

We also investigated the effect of different choices for one pipeline *S-S* under one simulation condition (0.5, 0.4, 0.1) for (P_G1_, P_G2_, P_G3_) shown in Table 3. We evaluated a total of six combinations, 2 *type* options (*“ratio”* and *“iterate”*) in the “estimateSizeFactors” function × 3 *fitType* options (*“parametric”*, *“local”*, and *“mean”*) in “estimateDispersions” function, provided in DESeq2. Overall, we found that different choices for *fitType* options had more impact than those of *type* options. Moreover, the use of *fitType* = *“parametric”* had the highest AUC values (82.01 and 81.91 % with *type = “ratio”* and *“iterate”*, respectively), followed by the uses of *“local”* (81.53 and 81.31 %), and *“mean”* (76.02 and 75.84 %). Since the best practice (i.e., the use of *type* = *“ratio”* and *fitType* = *“parametric”*) is the default in *S-S* (i.e., DESeq2), the choices should be left unchanged. Similar to the above described for *EEE-E*, TCC does not allow these options to be changed when performing *SSS-S*, which can be recommended for multi-group data without replicates. These results indicate that, as expected, suggested options should basically be used. The AUC values for these combinations are given in Additional file 11.

## Conclusion

We evaluated 12 pipelines for DE analysis of multi-group RNA-seq count data. Second to two-group comparison, this experimental design has arguably been performed well in practice. To our knowledge, the current evaluation is the first comprehensive study on multi-group count data. Our main findings can be summarized as follows:

First, the idea of DEGES implemented in TCC can be applied to multi-group data. We confirmed that the AUC values for the three DEGES-based pipelines (*EEE-E*, *DDD-D*, and *SSS-S*) were higher overall than the corresponding non-DEGES-based pipelines: *E-E* (edgeR), *D-D* (DESeq), and *S-S* (DESeq2), respectively (Table 1).

Second, choosing DEG identification method *Z* in the DEGES-based pipeline *XYX*-*Z* is critical for obtaining good DE results. For *Z* in the pipeline *XYX*-*Z*, using *E* (the DEG identification method provided in edgeR; Table 2) and *S* (provided in DESeq2; Table 3) when analyzing three-group data with and without replicates, respectively, gave higher AUC values than the others.

Third, to analyze three-group data with replicates, we recommend using either *DED-E* or *EEE-E* (Table 2). Both pipelines can easily be performed by using the TCC package. While *DED-E* showed the highest AUC values under the interrogated pipelines and simulation conditions, the difference between *DED-E* and the second best pipeline *EEE-E* can practically be negligible. Since *EEE-E* is the natural extension of a DEGES-based pipeline for edgeR, using *EEE-E* would be the best practice. However, note that two Bayesian pipelines (baySeq and EBSeq) perform comparably to or better than the GLM-based pipelines (edgeR, DESeq, and DESeq2) when a number of replicates are available (Additional file 2). In particular, EBSeq consistently outperformed *EEE-E* under some simulation conditions (Nrep = 9 and P_DEG_ = 5 %; Sheet 2 in Additional file 2), suggesting that the DEGES-based pipeline based on EBSeq could produce a more accurate ranked gene list. Although these Bayesian pipelines tend to come at the cost of a huge computation time, their implementation and evaluation are the next important tasks.

Fourth, to analyze three-group data without replicates, we recommend using either *EDE-S* or *SSS-S* (Table 3). Both pipelines can easily be performed by using the TCC package. While *EDE-S* showed the highest AUC values under the interrogated pipelines and simulation conditions, the difference between *EDE-S* and the second best pipeline *SSS-S* can be practically negligible. Since *SSS-S* is the natural extension of a DEGES-based pipeline for DESeq2, using *SSS-S* would be the best practice. Note that our previous recommendation for analyzing two-group data without replicates was to use *DDD-D* and that this conclusion was obtained only by evaluating a total of eight competing pipelines (*D-D*, *DDD-D*, *EDE-D*, *EbE-D*, *D-b*, *DDD-b*, *EDE-b*, and *EbE-b*, where “*b*” denotes baySeq). We expect the DESeq2-related pipelines (i.e., *EDE-S* and *SSS-S*) would be recommended for analyzing two-group data without replicates as an updated guideline. The comprehensive evaluation should, of course, be performed as one of the next tasks.

Fifth, the results of DE analysis (including existence or non-existence of DEGs) can roughly be estimated by the hierarchical dendrogram of sample clustering for the raw count data (Table 4; Additional files 6, 8, and 9). The dendrogram of sample clustering tells us some useful information about the DE results. The real count data we used here have 18.5 ~ 45.7 % of DEGs at the 5 % FDR threshold (Additional file 8). In our experience, such results (i.e., existence of large numbers of DEGs) have frequently been obtained when individual groups (G1, G2, and G3) form distinct sub-clusters where each sub-cluster consists only of members in each group (Additional file 6). In other words, if members in each sub-cluster originate from plural groups, no or few DEGs would be obtained as the DE result for such indistinct data. Of course, it is critical to employ appropriate choices for the distance metric and filtering of low count data for obtaining a robust dendrogram. While we employed the default options (“1 - Spearman correlation coefficient” as a distance and the use of *unique* expression patterns as an objective filtering) in the clustering function (“clusterSample”) provided in TCC, further evaluation should also be performed.

We speculate that the current recommendations made from the three-group comparative study can be applied to data consisting of three or more groups. While our preliminary analysis for four- and five-group simulation data has produced similar results to the current study, comprehensive evaluations are the next tasks.

## Methods

All analyses were performed using R (ver. 3.2.0 pre-release) [19] and Bioconductor [20].

### Simulation data

The three-group simulation data analyzed here were produced using the “simulateReadCounts” function in TCC. The variance (*V*) of the NB distribution can generally be modeled as *V* = *μ* + *Φμ*^2^. The empirical distribution of read counts for producing the mean (*μ*) and dispersion (*Φ*) parameters of the NB model was obtained from *Arabidopsis* data (three BRs for both the treated and non-treated samples) in [51]. The output of the “simulateReadCounts” function is stored in the TCC class object with information about the simulation conditions and is therefore ready-to-analyze.

### Real data

The real count dataset (“suppTable1.xls”) was obtained from the supplementary website of [50]. The raw count matrix consisting of 20,689 genes × 36 samples (=3 species × 2 sexes × 3 BRs × 2 technical replicates) were collapsed by summing the data of technical replicates, giving a reduced number of columns in the count matrix (i.e., 18 samples; 3 species × 2 sexes × 3 BRs). The three-group comparison of this dataset was performed by ignoring the sex differences (i.e., males or females). The relationship of sample names between the original and current study can be seen in Additional file 6.

### Differential expression analysis using individual packages

Gene lists ranked in accordance with the level of DE are pre-required for calculating AUC values. The input data for DE analysis using all R packages are the raw count data where each row indicates the gene (or transcript), each column indicates the sample (or library), and each cell indicates the number of reads mapped to the gene in the sample. The versions of major R packages were TCC ver. 1.7.15, edgeR ver. 3.8.5, DESeq ver. 1.18.0, DESeq2 ver. 1.6.3, limma ver. 3.22.1, samr ver. 2.0, PoissonSeq ver. 1.1.2, baySeq ver. 2.0.50, and EBSeq ver. 1.6.0.

All the DEGES-based pipelines *X*-(*Y*-*X*)_*n*_-*Z* or *XYX*-*Z* were performed using the TCC package. This pipeline includes *EEE-E*, *DED-E*, *EDE-E*, *DDD-E*, *EEE-D*, *DED-D*, *EDE-D*, *DDD-D*, *SSS-S*, *EEE-S*, *DED-S*, *EDE-S*, and *DDD-S*. Four other non-DEGES-based pipelines *X-Z* (*D-E*, *E-D*, *E-S*, and *D-S*) were also performed using this package, since they were the hybrid ones originally implemented in different packages. These DEGES-based and non-DEGES-based pipelines were performed using two functions: “calcNormFactors” for *X* (and *Y*) and “estimateDE” for *Z*, in the TCC package. For the DEGES-based pipelines *X*-(*Y*-*X*)_*n*_-*Z*, the options for *X*, *Y*, and *n* in the “calcNormFactors’ function correspond to *norm.method*, *test.method*, and *iteration*, respectively. The *E*, *D*, and *S* for *X* correspond to *norm.method = “tmm”*, *“deseq”*, and *“deseq2”*, respectively. The *E*, *D*, and *S* for both *Y* and *Z* correspond to *test.method = “edger”*, *“deseq”*, and *“deseq2”*, respectively. For *n* in the DEGES-based pipelines *X*-(*Y*-*X*)_*n*_-*Z*, the *iteration = 3* was used as recommended in [12]. For example, the *DED-S* pipeline was performed using the “calcNormFactors” function with *norm.method = “deseq”*, *test.method = “edger”*, and *iteration = 3* options, followed by the “estimateDE” function with *test.method = “deseq2”* option (see Additional file 4). The non-DEGES-based pipelines *X-Z* as *X*-(*Y*-*X*)_*0*_-*Z* were accomplished by applying *iteration = FALSE*. The genes were ranked in ascending order of the *p*-values. The *p*-value adjustment for the multiple-testing problem was performed using the “p.adjust” function with *method = “BH”* option (Benjamini-Hochberg FDR calculation).

The two functions in TCC internally use individual functions provided by one (or two) of the three other packages (edgeR, DESeq, and DESeq2) in accordance with the specific choices (i.e., *“tmm”*, *“edger”*, *“deseq”*, and *“deseq2”*) of options in TCC. The options used for individual functions in those three packages, internally used in TCC, are the same as those suggested in the original packages. Accordingly, three pipelines (i.e., *E-E*, *D-D*, and *S-S*) as the *default* procedures in edgeR, DESeq, and DESeq2 can also be performed using TCC. For example, the *S-S* pipeline in TCC can be performed using the “calcNormFactors” function with *norm.method = “deseq2”*, *test.method = NULL*, and *iteration = FALSE* options, followed by the “estimateDE” function with *test.method = “deseq2”* option (see Additional files 1 and 4). Although we did not employ TCC for the three pipelines in the current evaluation, researchers can easily learn what is done in TCC by comparing the corresponding original procedures described below.

Two pipelines, *E-E* (the same as the default edgeR procedure) and edgeR_robust, were performed using the edgeR package. The *E-E* pipeline for analyzing count data with replicates was performed using the following functions: “DGEList”, “calcNormFactors” with the *method = “TMM”* option, “estimateGLMCommonDisp” with *method = “CoxReid”* option, “estimateGLMTrendedDisp” with *method = “auto”* option, “estimateGLMTagwiseDisp”, “glmFit”, and “glmLRT” with *test = “chisq”* option. When analyzing count data without replicates, the “estimateGLMCommonDisp” function with three options (*method = “deviance”*, *robust = TRUE*, and *subset = NULL*) was used and two functions (“estimateGLMTrendedDisp” and “estimateGLMTagwiseDisp”) were not used, as suggested. The edgeR_robust method was performed using the following functions: “DGEList”, “calcNormFactors” with *method = “TMM”* option, “estimateGLMRobustDisp” with *prior.df = 10*, *maxit = 6*, and *record = FALSE* options, “glmFit”, and “glmLRT” with *test = “chisq”* option*.* The gene ranking and *p*-value adjustment procedure were performed the same way as described above.

The pipeline *D-D* was performed using the DESeq package. The *D-D* for analyzing data with replicates was performed using the following functions: “newCountDataSet”, “estimateSizeFactors” with *locfunc = median* option, “estimateDispersions” with *method = “pooled”*, *sharingMode = “maximum”*, and *fitType = “parametric”* options, and “fitNbinomGLMs”. When analyzing data without replicates, the “estimateDispersions” function with the following options was used as suggested: *method = “blind”* and *sharingMode = “fit-only”*. The genes were ranked in ascending order of the *p*-values. The *p*-value adjustment for the multiple-testing problem was performed using the “p.adjust” function with *method = “BH”* option (Benjamini-Hochberg FDR calculation).

The pipeline *S-S* in the DESeq2 package was performed using the following functions: “DESeqDataSetFromMatrix”, “estimateSizeFactors” with *type = “ratio”* option, “estimateDispersions” with *fitType = “parametric”* option, and “nbinomLRT” with *modelMatrixType = “standard’* option. The genes were ranked in ascending order of the *p*-values. Since this package provides adjusted *p*-values, the number of DEGs satisfying the 5 % FDR threshold was obtained using the values.

The pipeline voom in the limma package was performed using the following functions: “DGEList”, “calcNormFactors” with *method = “TMM”* option in edgeR, “voom”, “lmFit”, “eBayes”, and “topTable”. The gene ranking was performed using the resultant *p*-values. Since this package provides adjusted *p*-values, the number of DEGs satisfying the 5 % FDR threshold was obtained using the values.

The pipeline SAMseq in the samr package was performed using the “SAMseq” function with the following options: *nperms = 100*, *nresamp = 20*, *resp.type = “Multiclass”*, and *fdr.output = 1.0*. Since this package only provides adjusted *p*-values, the gene ranking was performed using the adjusted *p*-values.

The pipeline PoissonSeq was performed by using the “PS.Main” function with *npermu = 500* option. The gene ranking was performed using the resultant *p*-values. Since this package provides adjusted *p*-values, the number of DEGs satisfying the 5 % FDR threshold was obtained using the values.

The pipeline baySeq was performed using the following functions: “new”, “getLibsizes” with *estimationType = “edgeR”* option, “getPriors.NB” with *samplesize = 5000* and *estimation = “QL”* options, “getLikelihoods” with *pET = “BIC”* option, and “topCounts”. Since this package only provides adjusted *p*-values, the gene ranking was performed using the values. The *ordering* information in the output of the “topCounts” function was used for classifying the expression patterns of genes.

The pipeline EBSeq was performed using the following functions: “GetPatterns”, “MedianNorm”, “EBMultiTest” with three options (*maxround = 5*, *Qtrm = 1.0*, and *QtrmCut = −1*), and “GetMultiPP”. There are five expression patterns to consider when comparing three-group data. The “EBMultiTest” function was performed with the consideration of all the five possible patterns. The posterior probability obtained from the “non-DEG” pattern was used as a surrogate estimate for the adjusted *p*-values. The gene ranking was performed using the values. The *MAP* information in the output of the “GetMultiPP” function was used for classifying the expression patterns of genes.

## Additional files

Additional file 1:
**R code for obtaining simulation results with replicates (Table** 1 **).** After execution of this R-code with default parameter settings, the average AUC values of 100 trials under the following conditions shown in Table 1 can be obtained: P_DEG_ = 5 %, (0.5, 0.4, 0.1) for (P_G1_, P_G2_, P_G3_), and Nrep = 3. The results in Additional file 2 can also be obtained by changing the parameter Nrep to be 6 or 9. (R 12 kb) Additional file 2:
**Results for simulation data with replicates (mainly Nrep = 6 and 9).** Average AUC values (%) of 100 trials are shown for a total of 12 pipelines for three-group simulation data, where each group has six (Nrep = 6; **Sheet 1**) and nine (Nrep = 9; **Sheet 2**) BRs. **Sheet 3**: Average partial AUC values (%) of 20 trials with (1 - specificity) < 0.1. **Sheet 4**: Average computation times (in seconds) of 20 trials. The times were measured on a Windows system (Windows 7 Professional, Intel(R) Core(TM) i5-2540 M CPU, 2.60 GHz, and 8 GB memory). **Sheet 5**: Average AUC values (%) of 20 trials where the fold-changes for DEGs were randomly sampled from “1.2 + a gamma distribution with shape = 2.0 and scale = 0.5”. Other legends are the same as in Table 1. (XLSX 24 kb) Additional file 3:
**R code for obtaining simulation results with replicates (Table** 2 **).** After execution of this R-code with default parameter settings, the average AUC values of 100 trials under the following conditions shown in Table 2 can be obtained: P_DEG_ = 5 %, (0.5, 0.4, 0.1) for (P_G1_, P_G2_, P_G3_), and Nrep = 3. (R 4 kb) Additional file 4:
**R code for obtaining simulation results without replicates (Table** 3 **).** After execution of this R-code with default parameter settings, the average AUC values of 100 trials under the following conditions shown in Table 3 can be obtained: P_DEG_ = 25 %, (0.5, 0.4, 0.1) for (P_G1_, P_G2_, P_G3_), and Nrep = 1. (R 8 kb) Additional file 5:
**R code for obtaining results of Blekhman’s count data.** After execution of this R-code, full results of real data analysis can be obtained. (R 21 kb) Additional file 6:
**Dendrogram of average-linkage hierarchical clustering for the Blekhman’s count data.** Results of sample clustering are shown: (a) a raw count dataset consisting of 36 samples, (b) a collapsed data consisting of 18 samples, and (c) the same data as (b) but with different sample labels. The clustering was performed using the “clusterSample” function with default options provided in TCC. (PPTX 62 kb) Additional file 7:
**Dendrogram of average-linkage hierarchical clustering for 12 ranked gene lists.** Twelve ranked gene lists used for constructing the dendrogram were obtained from the analysis of the simulation data under the following conditions: P_DEG_ = 5 %, (0.5, 0.4, 0.1) for (P_G1_, P_G2_, P_G3_), and Nrep = 9. The clustering was performed using the “clusterSample” function with distances defined as (1 - Spearman’s rank correlation coefficient). EBSeq showed the highest AUC values (=96.83 %) in this simulation trial, followed by *EEE-E* (96.45 %), *E-E* (96.42 %), *DDD-D* (96.35 %), *D-D* (96.31 %), baySeq (96.21 %), edgeR_robust (95.13 %), *S-S* (94.54 %), *SSS-S* (94.43 %), PoissonSeq (94.07 %), voom (92.70 %), and SAMseq (92.23 %). (PNG 6 kb) Additional file 8:
**Comparison of DEGs obtained from individual pipelines for the Blekhman’s count data.** **Sheet 1**: Numbers of DEGs satisfying the 5 % FDR threshold and the overlaps between all pairs of pipelines are shown. The presentation method is the same as in table 1 in [28]: the numbers on the diagonal are highlighted in bold. **Sheet 2**: The corresponding Jaccard coefficients are shown. (XLSX 13 kb) Additional file 9:
**Classification of expression patterns for DEGs (based on EBSeq).** EBSeq defines a total of five possible patterns (Patterns 1 ~ 5). DEGs (satisfying 5 % FDR threshold) identified by individual pipelines were assigned to one of the five possible patterns. (XLSX 11 kb) Additional file 10:
**Percentages of Overlapping Genes (POGs) between ranked gene lists for 12 pipelines.** POG values for any numbers of top-ranked genes for individual pipelines are shown. Legends are basically the same as in Fig. 2. (PPTX 151 kb) Additional file 11:
**Average AUC values for simulation data with various options.** Average AUC values of 100 trials are shown. The suggested (or default) options and the highest AUC values are in bold. **Sheet 1**: *E-E* (edgeR), **Sheet 2**: *D-D* (DESeq), **Sheet 3**: *S-S* (DESeq2). (XLSX 12 kb)

## Abbreviations

AUC: The area under the curve
BB: Beta-binomial (distribution or model)
BR: Biological replicate
DE: Differential expression
DEG: Differentially expressed genes
DEGES: DEG elimination strategy
FDR: False discovery rate
GLM: Generalized linear model
HS: *Homo sapiens*
NB: Negative-binomial (distribution or model)
POG: Percentages of overlapping genes
PT: *Pan troglodytes*
RM: *Rhesus macaques*
TMM: Trimmed mean of M values (method)
TbT: the TMM-baySeq-TMM pipeline
TCC: Tag Count Comparison
