# Supplementary figures and images for: Evaluation of methods for differential expression analysis on multi-group RNA-seq count data
Source: BMC Bioinformatics. 2015 Nov 4;16:361. doi: 10.1186/s12859-015-0794-7 (PMC4634584; doi:10.1186/s12859-015-0794-7)

## Slide 1
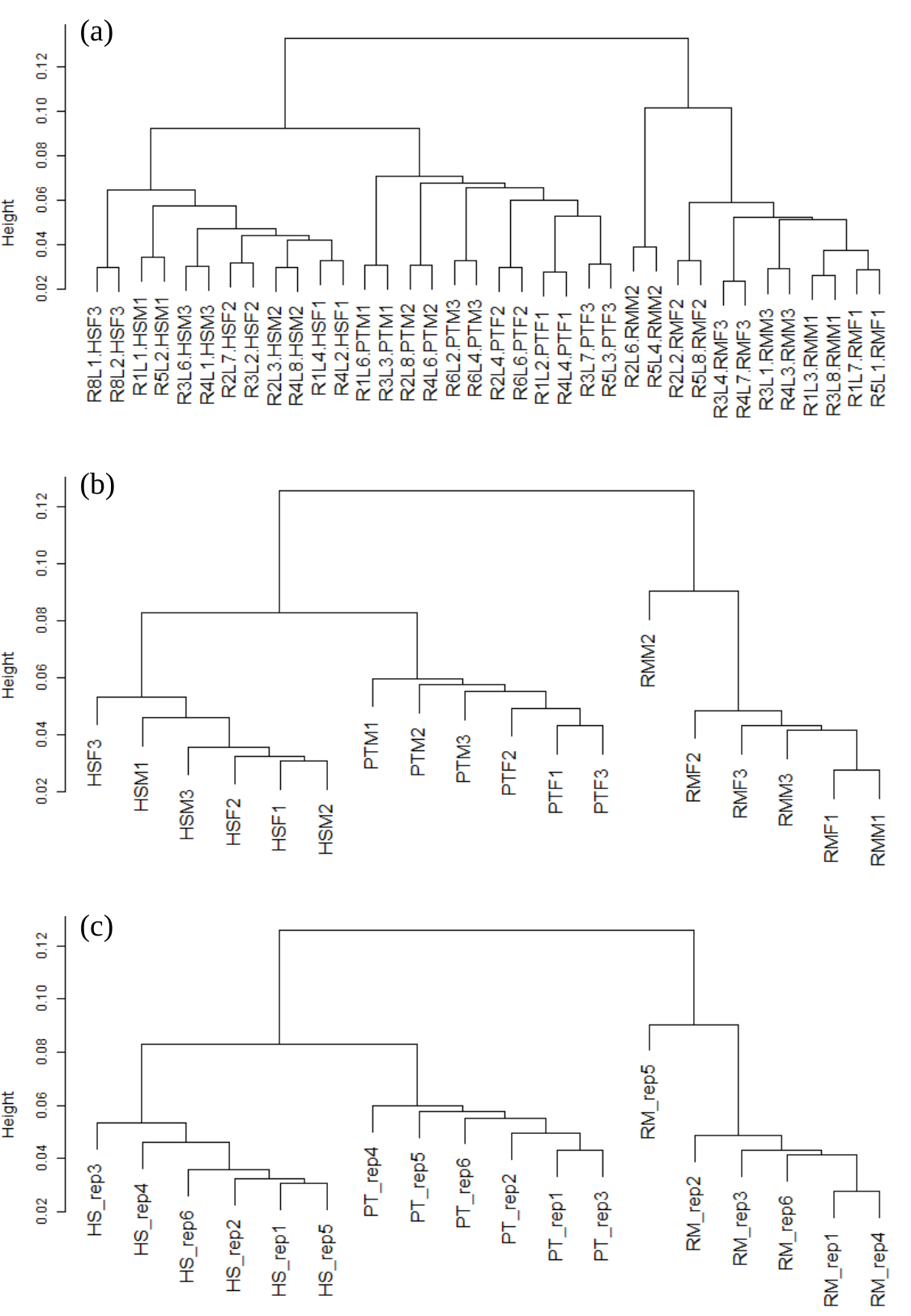

(a)
(b)
(c)

Supplement: Additional file 6: — Dendrogram of average-linkage hierarchical clustering for the Blekhman’s count data. Results of sample clustering are shown: (a) a raw count dataset consisting of 36 samples, (b) a collapsed data consisting of 18 samples, and (c) the same data as (b) but with different sample labels. The clustering was performed using the “clusterSample” function with default options provided in TCC. (PPTX 62 kb) [file 12859_2015_794_MOESM6_ESM.pptx]

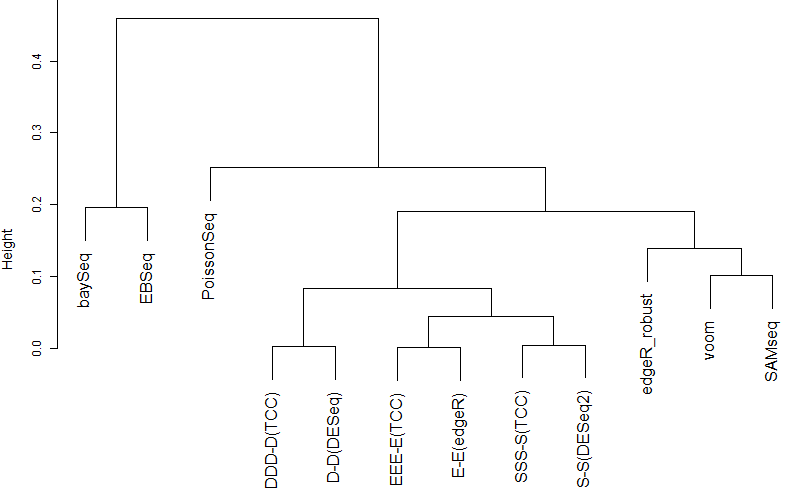

Supplement: Additional file 7: — Dendrogram of average-linkage hierarchical clustering for 12 ranked gene lists. Twelve ranked gene lists used for constructing the dendrogram were obtained from the analysis of the simulation data under the following conditions: PDEG = 5 %, (0.5, 0.4, 0.1) for (PG1, PG2, PG3), and Nrep = 9. The clustering was performed using the “clusterSample” function with distances defined as (1 - Spearman’s rank correlation coefficient). EBSeq showed the highest AUC values (=96.83 %) in this simulation trial, followed by EEE-E (96.45 %), E-E (96.42 %), DDD-D (96.35 %), D-D (96.31 %), baySeq (96.21 %), edgeR_robust (95.13 %), S-S (94.54 %), SSS-S (94.43 %), PoissonSeq (94.07 %), voom (92.70 %), and SAMseq (92.23 %). (PNG 6 kb) [file 12859_2015_794_MOESM7_ESM.png]

## Slide 1
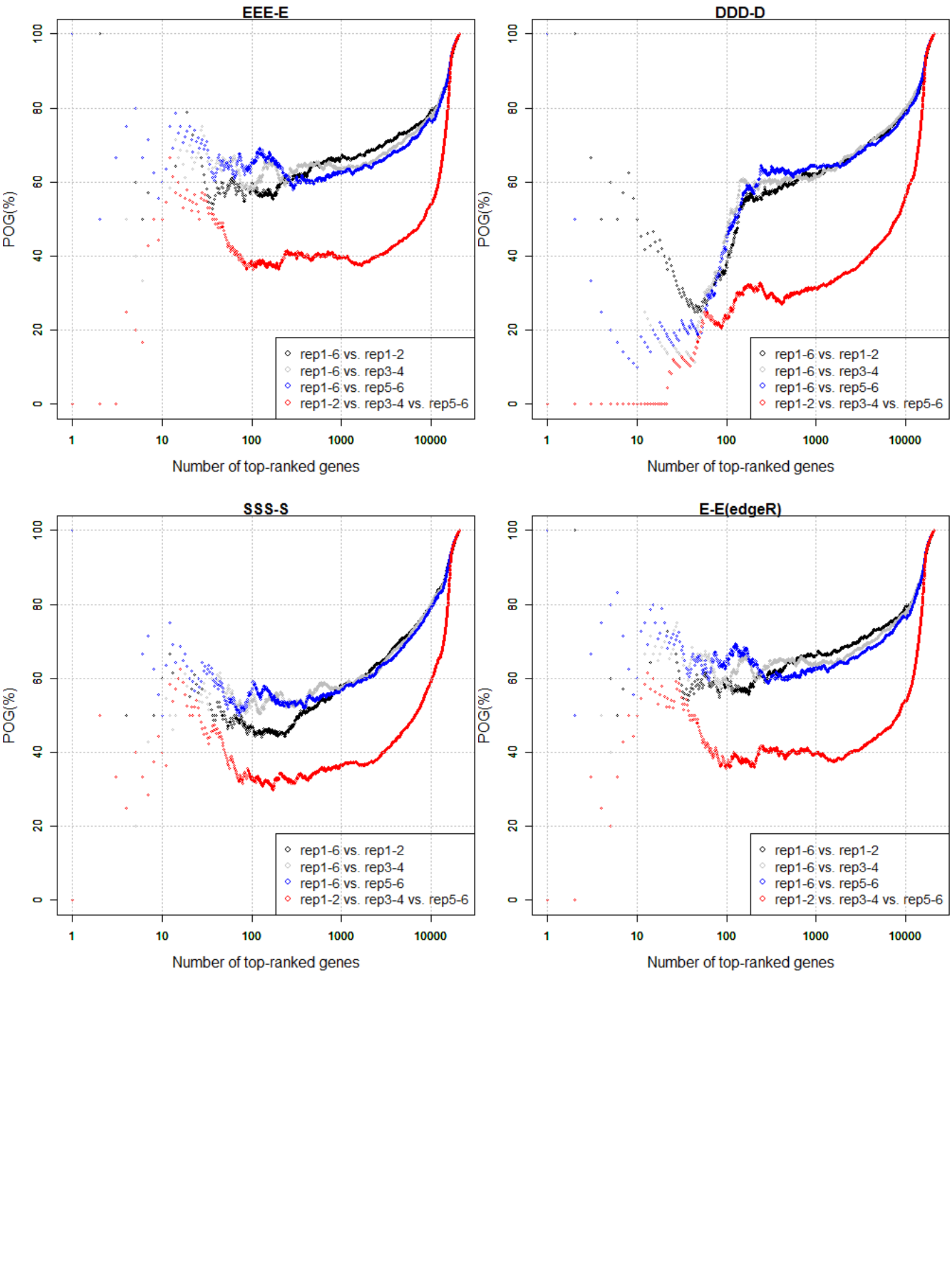

## Slide 2
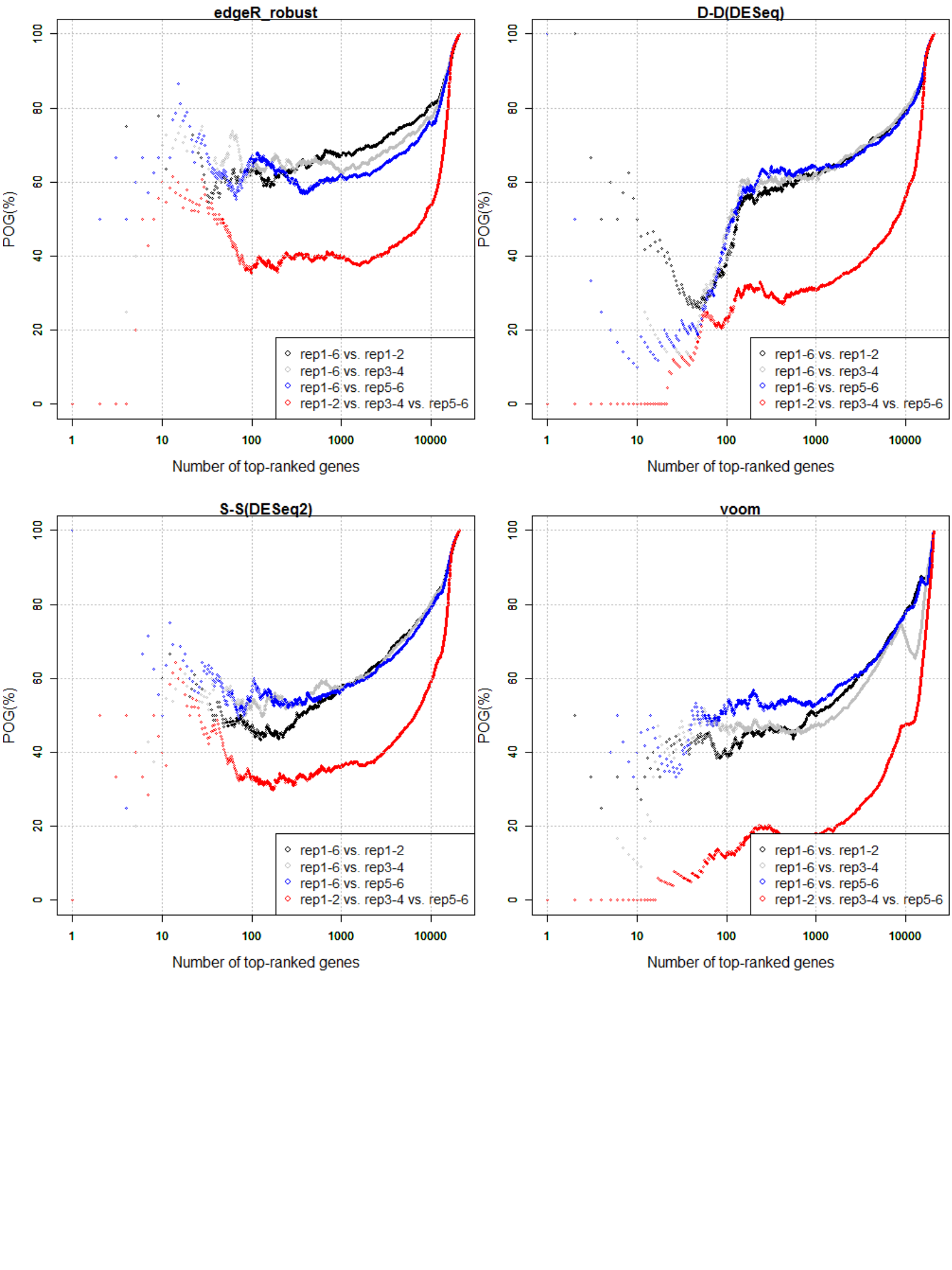

## Slide 3
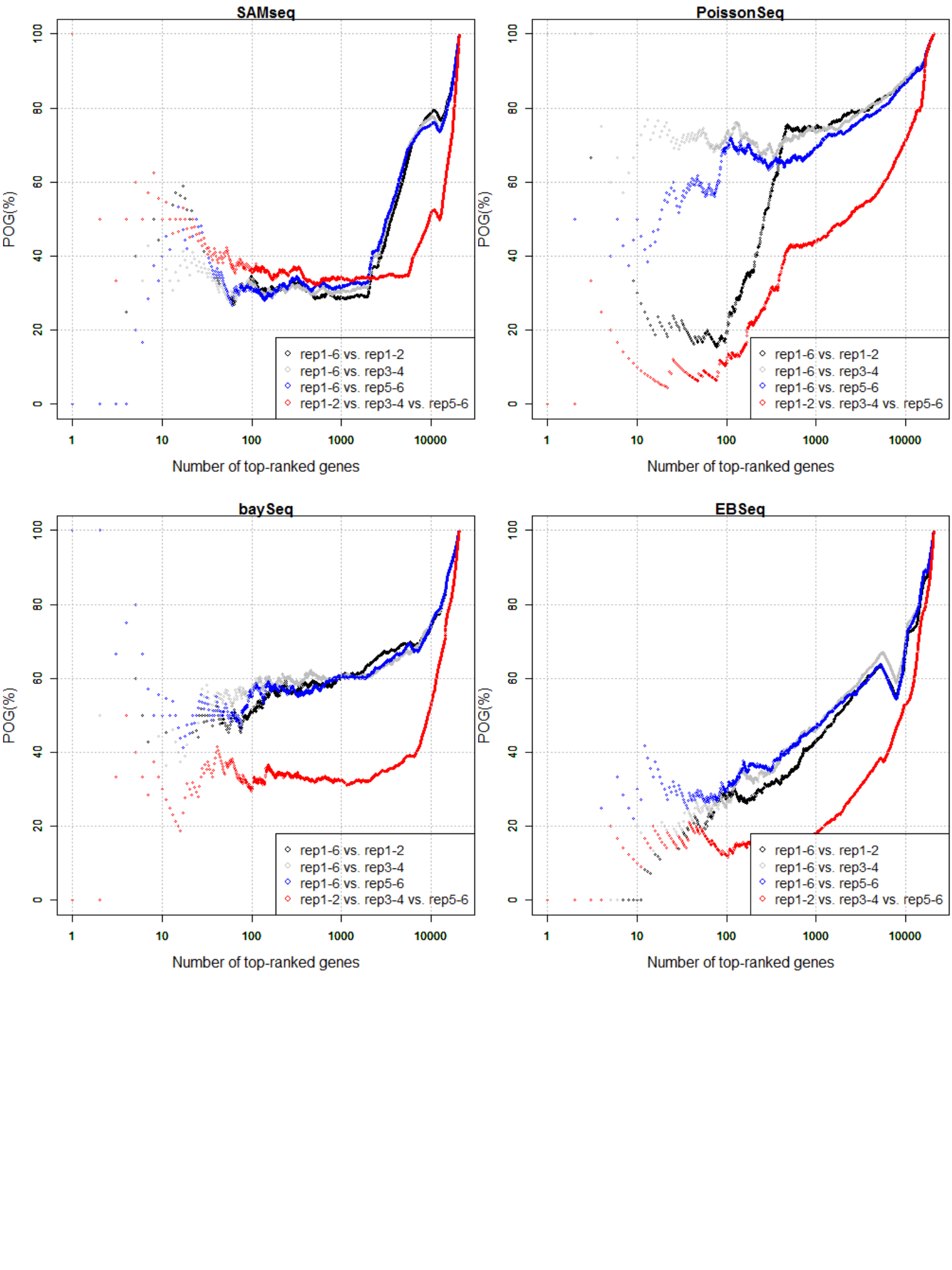

Supplement: Additional file 10: — Percentages of Overlapping Genes (POGs) between ranked gene lists for 12 pipelines. POG values for any numbers of top-ranked genes for individual pipelines are shown. Legends are basically the same as in Fig. 2. (PPTX 151 kb) [file 12859_2015_794_MOESM10_ESM.pptx]
